# Depression and Anxiety following Coronary Artery Bypass Graft: Current Indian Scenario

**DOI:** 10.1155/2016/2345184

**Published:** 2016-02-29

**Authors:** Suprakash Chaudhury, Rajiv Saini, Ajay Kumar Bakhla, Jaswinder Singh

**Affiliations:** ^1^Department of Psychiatry, Pravara Institute of Medical Sciences (Deemed University), Loni, Maharashtra 413736, India; ^2^Department of Psychiatry, AFMC, Pune, Maharashtra 411040, India; ^3^Department of Psychiatry, Rajendra Institute of Medical Sciences, Ranchi, Jharkhand 834009, India; ^4^Department of Cardiothoracic Surgery, MH CTC, Pune, Maharashtra 411040, India

## Abstract

Epidemiological studies have shown a high prevalence of coronary artery disease among the Indian Population. Due to increasing availability and affordability of tertiary care in many parts of India, carefully selected patients undergo coronary artery bypass surgery to improve cardiac function. However, the procedure is commonly associated with depression and anxiety which can adversely affect overall prognosis. The objective of this review is to highlight early identifiable symptoms of depression and anxiety following coronary artery bypass graft (CABG) in Indian context so as to facilitate prompt intervention for better outcome. The current review was able to establish firm evidence in support of screening for depression and anxiety following CABG. Management of depression and anxiety following CABG is briefly reviewed.

## 1. Introduction 

World Health Organization (WHO) describes health as a state of complete physical, mental, and social well-being and not merely an absence of disease or infirmity. Psychosomatic medicine acts as a bridge between psychiatry and other medical disciplines. Conceptually, the mind-body link has always fascinated medical man as ultimate acknowledgement of good treatment will eventually be appreciated by the mind and not the body.

However, psychiatric care has always been looked down upon as being meant for those who are inferior or mentally weak [1]. The situation is prevalent in all societies owing to stigma and discrimination towards mental illness and the mentally ill [2]. The practice often leads to denial of essential medical care with adverse outcomes. Psychiatrists, practicing on the interface of medicine and psychiatry, often find themselves creating new models of care to cater to local needs based on available resources. The issue has been discussed in detail wherein authors describe benefits of holistic medical care with active collaboration of psychiatrist and the primary care physician [3].

Vascular psychiatry is a newly emerging concept highlighting the need for psychiatric intervention in patients suffering from diseases of blood vessels [4]. It is well known that cardiovascular and cerebrovascular syndromes yield highest psychiatric morbidity and mortality. In daily practice, psychiatrists commonly encounter vascular syndromes, such as vascular depression, vascular cognitive impairment, and depression in heart disease. More often than not, psychiatric and vascular disorders occur together indicating common underlying etiopathological mechanisms [5]. Further, their association extends well into the immediate and long term care. These examples serve as innovative ways to collaborate and integrate comprehensive health care.

## 2. Coronary Artery Disease and Psychopathology 

Coronary artery disease is the leading cause of morbidity and mortality worldwide. For more than 15 years, WHO has been sounding an alarm on the rapidly rising burden of cardiovascular disorders. The reported prevalence of coronary artery disease (CAD) in adult surveys has risen 4-fold over the last 40 years to a present level of around 10% [6, 7]. It is the leading cause of death and disability worldwide. The incidence and prevalence in Indian population may be higher because of sociodemographic reasons. The recent past has been witness to some exciting advancements in cardiac care with emphasis on prevention, early detection, and therapeutic procedures [8].

During early stages, management of CAD includes dietary and life style modification, lipid lowering agents, blood pressure monitoring, glycemic control, and antiplatelet agents. As the disease progresses, these measures are not sufficient to maintain a satisfactory quality of life. Coronary angioplasty and coronary artery bypass graft surgery (CABG) offer promise of improved quality of life in such cases though their indications undergo revision in pace with latest recommendations. CABG is the commonest surgical method of management of CAD in India [9]. Over the years, refinement of surgical and anesthetic procedures has led to significant reduction in mortality and morbidity [10]. However, still a significant number of patients do have associated psychological morbidity which is disabling and distressing. Relationship of psychological symptoms with coronary heart disease has been well known since a long time [11]. It is important to note that psychological illness when comorbid with cardiac illness generally leads to poorer outcomes [12]. Depression has been found to be an independent prognostic factor for mortality, readmission, cardiac events, and lack of functional benefits 6 months to 5 years after CABG [13–16]. These observations highlight the need for integrating psychosocial interventions to provide holistic and effective management after CABG.

## 3. Cardiology, Neurology, and Psychiatry Interface 

The interface between heart and the mind is too strong to be negated. For reasoning to exist, a fine balance between the mind and the heart is needed for rational decision-making. When we speak of the mind we refer to the software of the hardware that we call brain. The integrity and the functionality of this software (mind) are based on optimal functioning of the underlying hardware (brain). Any insult to the structural integrity of the brain often gets translated into cognitive, emotional, motor, or sensory symptoms [17]. Motor, sensory, and neurocognitive domains are not under consideration in this paper though technically it is difficult to segregate them. The purpose of this review study is to highlight the role of early identification and management of emotional disorders that are encountered while caring for the patients undergoing CABG. We reviewed the literature for associations of CABG with negative emotions of depression or anxiety and their relationships with positive health related activities like regular drug adherence, healthy eating habits, regular exercise, and yoga.

## 4. Concept of Depression and Anxiety 

According to the International Classification for Diseases-tenth edition (ICD-10), depression is characterized by low mood and/or anhedonia (loss of interest in activities that once were pleasurable) that lasts for two weeks or more and is accompanied by significant functional impairment and somatic complaints of disturbed sleep, fatigue, body aches, digestive or sexual problems, and negative thoughts. Anxiety on the other hand refers to feeling of apprehension and unease. Anxiety has somatic, physiological, and cognitive components. Somatic component refers to digital tremors, palpitations, and sweaty palms. The physiological component refers to tachycardia, hyperventilation, muscular tension, and an irritable bladder. The cognitive component is that of worry which refers to undue fear of something untoward happening [18].

It is not uncommon to find both depression and anxiety to coexist on a continuum so much so that they are considered together as both impair one's quality of life and interfere significantly with the ability to think rationally. In fact, anxiety has often been described to be an integral component of depressive disorder and they respond to similar drugs to a large extent [19].

Researchers have tried to pinpoint the etiological basis of depression in cardiac illnesses and have implicated factors like hypercortisolemia, insulin resistance and sympathetic-parasympathetic tone dysregulation, reduced heart rate variability, hypothalamic-pituitary-adrenal axis (HPA) axis, and increased inflammatory factors like platelet factor 4, fibrinogen, and C-reactive protein. Unhealthy lifestyle like cigarette smoking, excessive alcohol intake, lack of physical exercise, poor medications adherence, and unhealthy diet may also be directly or indirectly contributing to the onset and progression of depression [20–22]. The etiopathogenesis of anxiety among patients of heart disease is less well understood. Threat perception and felt need for biological integrity have been consistently shown to have sympathetic nervous system upregulation with excessive catecholamine production [23]. Patients with CAD often have abnormally high levels of catecholamines, which can result in increased myocardial oxygen demand due to elevations in heart rate, blood pressure, and the rate of ventricular contraction. Additionally, additive effects of benzodiazepines, alcohol, and smoking also must be taken into account as such patients are often found to be abusing them [24].

## 5. Manifestations of Depression and Anxiety in Indian Subjects 

Though, the construct of Depression and anxiety is universally applicable as is the prevalence of these disorders, but cultural differences do exist as far as the description of the symptoms is concerned which may lead to underdiagnosis [25–28]. Visit to a local superspecialty hospital with more than 200 CABGs done per year revealed that only 5 cases were referred for psychiatric opinion because they had an earlier record of psychiatric treatment. To summarize, it is fair enough to accept that identification and referral pattern of patients suffering from psychiatric symptomatology are abysmally low in our population.

## 6. CABG: Indian Scenario

Studies on Indian immigrants and cross-sectional studies in India highlight high incidence of CAD in India [6, 7]. In absence of social security and state funding of medical care, only a fraction of them can actually afford superspecialty care like angioplasty or CABG. CABG was first performed in India in 1975 about 13 years after its advent in 1962. In the mid-1990s, some 10,000 CABG surgeries were being performed annually in India. Presently, the annual number is about 60000 according to industry sources [9, 29–31]. In the absence of a central registry, the exact numbers may not be known. There is no regularized health sector except in some metropolitan cities and health care is tightly compartmentalized. The majority of patients remain undiagnosed and those who are diagnosed have limited means of specialized care. It is also acknowledged that medical tourism is booming in this country and many tertiary care superspecialty hospitals cater to the rich who exclusively visit India for medical reasons. Many such hospitals offer medical package for a particular amount and the macro- and microeconomics determine the kind of medical care that will eventually be rendered to the patients [32]. Indian patients also have some other distinct peculiarities. These include younger age at presentation (average age 60 years), a high incidence of double (DVD) and triple vessel disease (TVD), diffuse involvement, distal disease, and significant left ventricular dysfunction at presentation [33, 34]. An angiographic study from Vellore in 1066 consecutive males admitted for CAD noted significant disease in 877 patients; of these, 55 percent were <50 yr of age, 34 percent were <45 yr of age, and 12 percent were below 40 yr of age. Although the mean age was 48 yr, TVD was more common (55%) than DVD (24%) and single vessel disease (24%) combined [30]. However, this data may not reflect true state of affairs as it comes from a tertiary care hospital. Another finding is that the majority of Indian patients also have many modifiable risk factors like high stress levels, smoking, hypertension, obesity, and diabetes [31, 35]. Such information opens a window of opportunity for collaborative intervention for long-term gains. There are several technical challenges, which cardiac surgeons in India have to face. These are chiefly related to small coronary vessels, arterial conduits, diffuse disease, and late presentation [9, 34]. In a recent study, authors noted that heart weight in Indians varied from 148 to 249 g while in the West the average weight of the heart in males is 300 g and that in females is 250 g [34]. Such smaller sized vessels pose difficulty during anastomosis and may result in early graft closure leading to higher mortality. Indians also tend to have diffuse CAD because of which vessels frequently require endarterectomy. The condition further predisposes to perioperative myocardial infarction and postoperative occlusion of bypass grafts [9, 33–38].

## 7. CABG and Psychopathology

Neuropsychiatric complications following CABG are well known ever since the procedure came into vogue. The range of these complications ranges from anxiety, depression, neurocognitive deficits, delirium, and cerebrovascular accident. The range varies from a conservative 2–4% to about 25–40% severe cases [39]. The scope of the current paper is restricted only to depression and anxiety and other effects like delirium; cerebrovascular and neurocognitive deficits are not being discussed here.

Depression and coronary artery disease are highly comorbid conditions with estimates of comorbidity from 14% to 47% [40, 41]. The causes of depression are no different from other causes of depression though it may appear that patient's depression is secondary to the diagnosis and will recover with surgery. The issue has been debated many times with clear finding that this is not the case. Though both depression and CAD may share same etiopathogenesis, they both need to be diagnosed and treated independently [21]. It is like a patient suffering abdominal trauma and fractured femur following an accident. Both conditions need attention for complete recovery. Preoperative depression is predictive of decreased cardiac symptom relief, quicker return of symptoms, more frequent rehospitalizations, and increased mortality in the immediate postoperative period [42–44].

Postoperative depression too is associated with delayed wound healing, higher infection rate, poor physical and emotional health, reduced pain threshold, and more adverse cardiac events like myocardial infarction and early death [45]. All these factors lead to poor overall quality of life and rising health costs.

Manifestation of anxiety in cardiac patients has been debated for some time and is often taken as a normal reaction. Some of the symptoms may closely mimic symptoms of CAD itself but an experienced clinician can easily make out the difference and understand the need to differentiate the two. Pathological anxiety manifests as a feeling of impending doom, excessive worrying thoughts of being disabled, persistent palpitations, generalized muscular tension with inability to relax, breathlessness, hyper vigilance, persistent headache, frequent urge to pass urine, butterflies in stomach, and persistent sleep disturbance. Frequently, such symptoms are either ignored or not asked/reported. However, they cause significant distress and may lead to adverse outcomes. It has been found to be unusually high for CABG patients while on the waiting list with an unknown surgery date [46]. Fear of dying before rather than during surgery has been highlighted as a pervasive and anxious preoccupation [47]. After the surgery, persistence of these symptoms is an ominous sign and may reflect poorer outcome. Incidence of anxiety was found to be more in younger than in older patients [44]. Following CABG, anxiety precipitates cardiac decompensation owing to higher autonomic arousal thus delaying healing and recovery. The most common anxiety disorders appear to be generalized anxiety disorder (GAD) and Panic Disorder with prevalence ranging from zero to 11%. Other anxiety disorders are Phobias (2.5–4.3%), Obsessive Compulsive Disorder (0.6–9%), and posttraumatic stress disorder (PTSD) (4–11%) [40].

## 8. Therapeutic Implications of Depression and Anxiety in CABG

Psychological intervention with cardiac patients reduces psychological pain, severe anxiety, hostility, and depression and thus improves quality of life as well. Common therapeutic approach seems particularly important keeping in view improved outcome and reduction in overall costs [48]. Presence and persistence of depression may have direct bearing on participation in cardiac rehabilitation and lifestyle modification program among CABG surgery patients. Similarly, persisting anxiety can be disabling and may further compromise recovery. A diverse range of behavioral and psychological RCT interventions have clearly demonstrated significant improvements in overall outcome and quality of life of such patients [49]. A recent Indian study highlighted the role of structured yoga therapy in improving the outcome of patients requiring CABG. It was the first time that a structured yoga program incorporating instruments like Hospital Anxiety Depression Scale (HADS), Perceived Stress Scale (PSS-14), and Positive and Negative Affect Scale (PANAS) was used. In a single blind fashion, the study was conducted on 1026 patients and positive effects were found in terms of Left Ventricular Ejection Fraction (LVEF), Body Mass Index (BMI), blood pressure and sugar control, depression, and anxiety symptoms [50]. SSRIs have proven safety and efficacy record and are generally the preferred pharmacological agents to be used in such cases [51]. An added benefit of these drugs is that they are equally efficacious for both depression and anxiety. In selected patients, it is fair enough to start the therapy at a low dose then escalate as per the response. American Heart Association (AHA) recommends that fair trial with two SSRIs should be given before switching on to other groups of antidepressants like serotonergic noradrenergic reuptake inhibitors like Bupropion [52]. Tricyclics antidepressants are effective in treating depression and anxiety, but their use has declined owing to their potential for cardiotoxicity. Since safer options are available in today's era, role of tricyclic antidepressants is limited. The line of management of both depression and anxiety is as per the guidelines laid for these disorders. Short courses are generally of limited clinical benefit due to likelihood of relapse. No consensus exists as far as the duration of such treatment is concerned but it is prudent to follow up the patient for at least six months after surgery and then review the treatment plan. The role of lifestyle modifications and behavioral treatments like yoga cannot be underestimated here as such strategies hold promise for long-term benefits. The recommendation is keeping in view with popular sentiment in this country. In Indian settings, patients are generally hesitant in reporting emotional distress and often hesitate in seeking emotional support. Busy clinicians may also miss subtle signs of emotional distress. Therefore, sensitive instruments in the form of questionnaires must be incorporated in the workup schedule. After discharge from the hospital, an information brochure containing early warning signs of emotional disorder can be given to the patient or care giver and they must be encouraged to clarify their queries during follow-up.

## 9. Conclusion

Coronary artery disease is the most important cause of morbidity and mortality in Indian subcontinent. There have been rapid advances in the care of those suffering its effects. Strong biological link between emotional state and coronary artery disease is well established. The paper has attempted to contextualize the findings in Indian setting with a view towards early identification and prompt intervention with established methods. The paper ends with a broad outline towards management of such patients from psychiatrist's perspective. A collaborative approach is likely to be of benefit for the patient and cost effective in the long run. It may be prudent to screen the patients during routine workup before and after surgery. Many patients may not be able to describe their symptoms in busy outpatient set-up. Under such conditions, patient education and awareness may be a useful strategy.

## References

[1] Jadhav S., Littlewood R., Ryder A. G., Chakraborty A., Jain S., Barua M. (2007). Stigmatization of severe mental illness in India: against the simple industrialization hypothesis. *Indian Journal of Psychiatry*.

[2] Kishore J., Gupta A., Jiloha R. C., Bantman P. (2011). Myths, beliefs and perceptions about mental disorders and health-seeking behavior in Delhi, India. *Indian Journal of Psychiatry*.

[3] Epstein L. A., Huffman J. C. (2009). Introduction. *Harvard Review of Psychiatry*.

[4] Ballard C., O'Sullivan M., Serra-Mestres J., Stewart R., Thomas A. Vascular psychiatry: the interface between vascular disease and mental disorders, and its clinical relevance.

[5] Barnes D. E., Alexopoulos G. S., Lopez O. L., Williamson J. D., Yaffe K. (2006). Depressive symptoms, vascular disease, and mild cognitive impairment. findings from the cardiovascular health study. *Journal of the American Medical Association*.

[6] Zachariah G., Harikrishnan S., Krishnan M. N. (2013). Prevalence of coronary artery disease and coronary risk factors in Kerala, South India: a population survey—design and methods. *Indian Heart Journal*.

[7] Rao M., Xavier D., Devi P. (2015). Prevalence, treatments and outcomes of coronary artery disease in Indians: a systematic review. *Indian Heart Journal*.

[8] Cohn W. E. (2010). Advances in surgical treatment of acute and chronic coronary artery disease. *Texas Heart Institute Journal*.

[9] Kaul U., Bhatia V. (2010). Perspective on coronary interventions & cardiac surgeries in India. *Indian Journal of Medical Research*.

[10] Erkut B., Dag O., Kaygin M. A. (2013). On-pump beating-heart versus conventional coronary artery bypass grafting for revascularization in patients with severe left ventricular dysfunction: early outcomes. *Canadian Journal of Surgery*.

[11] Herrmann-Lingen C. (2001). Anxiety and depression in cardiology patients: how to diagnose, how to treat?. *Herz*.

[12] Rugulies R. (2002). Depression as a predictor for coronary heart disease: a review and meta-analysis. *American Journal of Preventive Medicine*.

[13] Carney R. M., Freedland K. E., Steinmeyer B. (2008). Depression and five year survival following acute myocardial infarction: a prospective study. *Journal of Affective Disorders*.

[14] Mayou R. A., Gill D., Thompson D. R. (2000). Depression and anxiety as predictors of outcome after myocardial infarction. *Psychosomatic Medicine*.

[15] Lett H. S., Blumenthal J. A., Babyak M. A. (2004). Depression as a risk factor for coronary artery disease: evidence, mechanisms, and treatment. *Psychosomatic Medicine*.

[16] Frasure-Smith N., Lespérance F. (2005). Reflections on depression as a cardiac risk factor. *Psychosomatic Medicine*.

[17] Aben I., Verhey F. (2006). Depression after a cerebrovascular accident. The importance of the integration of neurobiological and psychosocial pathogenic models. *Panminerva Medica*.

[18] WHO (1992). ICD-10. Classification of mental and behavioural disorders. *Clinical Descriptions and Diagnostic Guidelines*.

[19] Clark L. A., Watson D. (1991). Tripartite model of anxiety and depression: psychometric evidence and taxonomic implications. *Journal of Abnormal Psychology*.

[20] Pratt L. A., Ford D. E., Crum R. M., Armenian H. K., Gallo J. J., Eaton W. W. (1996). Depression, psychotropic medication, and risk of myocardial infarction: prospective data from the Baltimore ECA follow-up. *Circulation*.

[21] Ravven S., Bader C., Azar A., Rudolph J. L. (2013). Depressive symptoms after CABG surgery: a meta analysis. *Harvard Review of Psychiatry*.

[22] Hasler G. (2010). Pathophysiology of depression: do we have any solid evidence of interest to clinicians?. *World Psychiatry*.

[23] Player M. S., Peterson E. L. (2011). Anxiety disorders, hypertension, and cardiovascular risk: a review. *International Journal of Psychiatry in Medicine*.

[24] Comeau N., Stewart S. H., Loba P. (2001). The relations of trait anxiety, anxiety sensitivity, and sensation seeking to adolescents' motivations for alcohol, cigarette, and marijuana use. *Addictive Behaviors*.

[25] Gautam S., Jain N. (2010). Indian culture and psychiatry. *Indian Journal of Psychiatry*.

[26] Avasthi A. (2014). Depression in primary care. Challenges and controversies. *Indian Journal of Medical Research*.

[27] Malhotra S., Chakrabarti S., Shah R. (2014). Development of a novel diagnostic system for a telepsychiatric application: a pilot validation study. *BMC Research Notes*.

[28] Ganguli M., Dube S., Johnston J. M., Pandav R., Chandra V., Dodge H. H. (1999). Depressive symptoms, cognitive impairment and functional impairment in a rural elderly population in India: a Hindi version of the geriatric depression scale (GDS-H). *International Journal of Geriatric Psychiatry*.

[29] Enas E. A., Singh V., Munjal Y. P. (2009). Recommendations of the second Indo-U.S. health summit on prevention and control of cardiovascular disease among Asian Indians. *Indian Heart Journal*.

[30] Saha K. K. (2014). Off pump coronary artery grafting in India. *Indian Heart Journal*.

[31] Kasliwal R. R., Kulshreshtha A., Agrawal S., Bansal M., Trehan N. (2006). Prevalence of cardiovascular risk factors in Indian patients undergoing coronary artery bypass surgery. *Journal of Association of Physicians of India*.

[32] Press Trust of India (2013). *India Ranks 3rd in Medical Tourism in Asia*.

[33] Indrayan A. Forecasting vascular disease cases and associated mortality in India. http://www.whoindia.org/LinkFiles.

[34] Brister S. J., Hamdulay Z., Verma S., Maganti M., Buchanan M. R. (2007). Ethnic diversity: South Asian ethnicity is associated with increased coronary artery bypass grafting mortality. *Journal of Thoracic and Cardiovascular Surgery*.

[35] Dani S., Sinha N., Bhargava B., Jain V., Reddy V. Y., Biswas P. (2007). Report of the coronary cardiac interventions registry of India the cardiological society of India for the year 2006. *Indian Heart Journal*.

[36] Dodge J. T., Brown B. G., Bolson E. L., Dodge H. T. (1992). Lumen diameter of normal human coronary arteries: influence of age, sex, anatomic variation, and left ventricular hypertrophy or dilation. *Circulation*.

[37] Edwards F. H., Carey J. S., Grover F. L., Bero J. W., Hartz R. S. (1998). Impact of gender on coronary bypass operative mortality. *Annals of Thoracic Surgery*.

[38] O'Connor N. J., Morton J. R., Birkmeyer J. D., Olmstead E. M., O'Connor G. T. (1996). Effect of coronary artery diameter in patients undergoing coronary bypass surgery. *Circulation*.

[39] Breuer A. C., Furlan A. J., Hanson M. R. (1983). Central nervous system complications of coronary artery bypass graft surgery: prospective analysis of 421 patients. *Stroke*.

[40] Tully P. J., Baker R. A. (2012). Depression, anxiety, and cardiac morbidity outcomes after coronary artery bypass surgery: a contemporary and practical review. *Journal of Geriatric Cardiology*.

[41] Utriyaprasit K., Moore S. (2005). Recovery symptoms and mood states in Thai CABG patients. *Journal of Transcultural Nursing*.

[42] Gallagher R., McKinley S. (2009). Anxiety, depression and perceived control in patients having coronary artery bypass grafts. *Journal of Advanced Nursing*.

[43] Nemati M. H., Astaneh B. (2011). The impact of coronary artery bypass graft surgery on depression and anxiety. *Journal of Cardiovascular Medicine*.

[44] Krannich J.-H. A., Weyers P., Lueger S., Herzog M., Bohrer T., Elert O. (2007). Presence of depression and anxiety before and after coronary artery bypass graft surgery and their relationship to age. *BMC Psychiatry*.

[45] Cserép Z., Székely A., Merkely B. (2013). Short and long term effects of psychosocial factors on the outcome of coronary artery bypass surgery. *Artery Bypass*.

[46] Koivula M., Tarkka M.-T., Tarkka M., Laippala P., Paunonen-Ilmonen M. (2002). Fear and anxiety in patients at different time-points in the coronary artery bypass process. *International Journal of Nursing Studies*.

[47] Fitzsimons D., Parahoo K., Richardson S. G., Stringer M. (2003). Patient anxiety while on a waiting list for coronary artery bypass surgery: a qualitative and quantitative analysis. *Heart and Lung*.

[48] Donohue J. M., Belnap B. H., Men A. (2014). Twelve-month cost-effectiveness of telephone-delivered collaborative care for treating depression following CABG surgery: a randomized controlled trial. *General Hospital Psychiatry*.

[49] Rollman B. L.,  Belnap M. S., Mazumdar S. (2095). Telephone-delivered collaborative care for treating post-CABG depression: a randomized controlled trial. *Journal of the American Medical Association*.

[50] Raghuram N., Parachuri V. R., Swarnagowri M. V. (2014). Yoga based cardiac rehabilitation after coronary artery bypass surgery: one-year results on LVEF, lipid profile and psychological states. A randomized controlled study. *Indian Heart Journal*.

[51] Chocron S., Vandel P., Durst C. (2013). Antidepressant therapy in patients undergoing coronary artery bypass grafting: the MOTIV-CABG trial. *Annals of Thoracic Surgery*.

[52] Hillis D. L., Smith P. K., Anderson J. L. (2011). 2011 ACCF/AHA guideline for coronary artery bypass graft surgery. A report of the American College of Cardiology Foundation/American Heart Association task force on practice guidelines. *Circulation*.

